# Thyroid Hormones, T3 and T4, in the Brain

**DOI:** 10.3389/fendo.2014.00040

**Published:** 2014-03-31

**Authors:** Amy C. Schroeder, Martin L. Privalsky

**Affiliations:** ^1^Department of Microbiology and Molecular Genetics, College of Biological Sciences, University of California Davis, Davis, CA, USA

**Keywords:** T4 thyronine, T3 thyronine, thyroid hormone receptor, brain, coregulator, deiodinase 2

## Abstract

Thyroid hormones (THs) are essential for fetal and post-natal nervous system development and also play an important role in the maintenance of adult brain function. Of the two major THs, T_4_ (3,5,3′,5′-tetraiodo-l-thyronine) is classically viewed as an pro-hormone that must be converted to T_3_ (3,5,3′-tri-iodo-l-thyronine) via tissue-level deiodinases for biological activity. THs primarily mediate their effects by binding to thyroid hormone receptor (TR) isoforms, predominantly TRα1 and TRβ1, which are expressed in different tissues and exhibit distinctive roles in endocrinology. Notably, the ability to respond to T_4_ and to T_3_ differs for the two TR isoforms, with TRα1 generally more responsive to T_4_ than TRβ1. TRα1 is also the most abundantly expressed TR isoform in the brain, encompassing 70–80% of all TR expression in this tissue. Conversion of T_4_ into T_3_ via deiodinase 2 in astrocytes has been classically viewed as critical for generating local T_3_ for neurons. However, deiodinase-deficient mice do not exhibit obvious defectives in brain development or function. Considering that TRα1 is well-established as the predominant isoform in brain, and that TRα1 responds to both T_3_ and T_4_, we suggest T_4_ may play a more active role in brain physiology than has been previously accepted.

## Introduction

Thyroid hormones (THs) are synthesized by the thyroid gland and are critical regulatory molecules with important roles in vertebrate physiology and development, including fetal and post-natal nervous system development and the maintenance of adult brain function (1, 2). The TH requirement for development is most apparent in the central nervous system (CNS) where severe TH deficiency in fetal and neonatal periods results in cretinism, a disease characterized by mental retardation, deafness, and ataxia; these consequences are irreversible if not treated soon after birth (3–5). Additionally, untreated hypothyroidism in the adult is associated with severe intellectual defects, abnormal balance and defects in fine motor skills, spasticity, and deafness (6). Correcting TH deficiencies is critical for normal brain development and function.

Thyroid hormones mediate CNS effects primarily through thyroid hormone receptors (TRs), members of the nuclear hormone receptor family (4, 7, 8). TRs bind to the DNA regulatory regions of target genes to activate or repress transcription through interactions with accessory proteins known as coregulators. There are two major THs, which bind to and activate TRs: T_3_ (3,5,3′-triiodo-l-thyronine) and T_4_ (3,5,3′,5′-tetraiodo-l-thyronine, also known as thyroxine). T_4_ differs from T_3_ by an additional iodine located at the 5′-position of the first thyroxine ring. T_3_ has been assumed to be the active form of TH, as T_3_ binds to TRs with a greater affinity than T_4_. In this model, T_4_ is thought to simply act as a pro-hormone, existing only to be circulated in the serum and converted at the tissue-level to T_3_ through an enzymatic reaction involving the removal of the 5′-iodine atom from T_4_ by local deiodinases (9, 10). Nonetheless, it is notable that most of the TH produced under normal conditions in the thyroid is secreted in the form of T_4_ and steady-state serum concentrations of T_4_ are many fold greater than those of T_3_ (11–14). Notably, iodine intake is important for the maintenance of both of these TH levels in circulation. In fact, during gestation and lactation in females, double the normal iodine intake is required to maintain adequate T_3_ and T_4_ in circulation to ensure normal fetal development (15, 16). Under conditions of low iodine intake, the serum T_3_/T_4_ ratio is somewhat increased reflecting the reduced abundance of iodine atoms (16). Although the ready availability of dietary iodized salt has largely eliminated these iodine deficiencies for school children in most developed countries today, these advances are often not adequate for pregnant and lactating women (17).

Indeed the primary TH crossing the adult blood–brain barrier (BBB) is believed to be T_4_; therefore, the adult brain may have access to sufficiently high levels of T_4_ to allow for direct binding to and transcriptional activation of TRs (18, 19). In fact, we know that both T_4_ and T_3_ binding by TRs lead to very similar structural changes in the receptor (12). Several reports have also shown that T_4_ exhibits non-genomic effects by interacting with integrin cell membrane receptors (20). These studies suggest that T_4_ may exhibit a greater role in physiology than merely acting as a pro-hormone. Therefore, the precise role of T_4_ as a pro-hormone and whether T_4_ might function directly as an active hormone in the CNS, remain incompletely answered questions.

## T_4_ Synthesis, Transport, and Availability in the Brain

**Figure 1:**
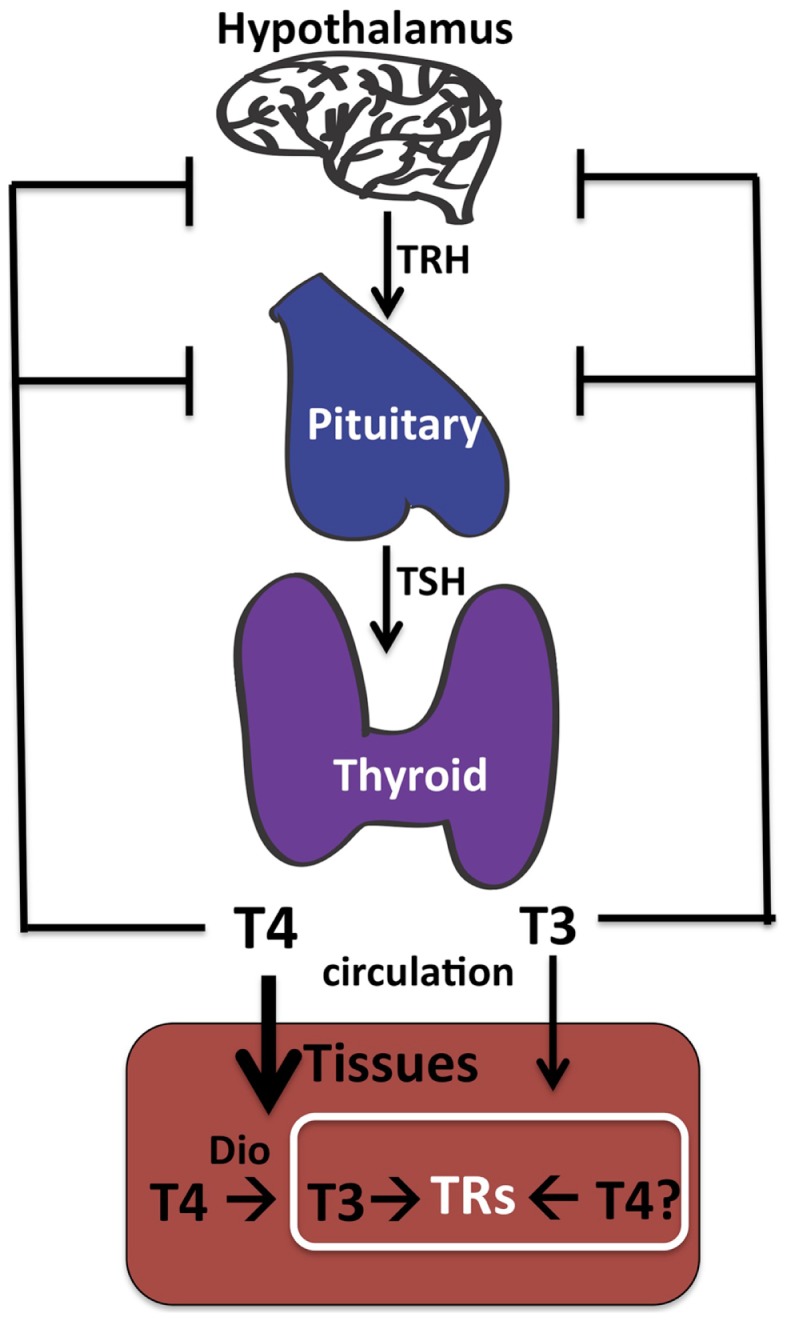
**Thyroid hormone synthesis**. The thyroid gland makes both T_4_ and T_3_, although T_4_ predominates. The hypothalamus senses low TH in the circulation and responds by stimulating synthesis and secretion of TRH (thyroid releasing hormone), which in turn circulates and stimulates synthesis and secretion of TSH (thyroid stimulating hormone) by the pituitary. Circulating TSH then increases T_4_ and T_3_ production by the thyroid and ultimately in the circulation. Tissue-specific deiodinases (“DIO”) are expressed in peripheral tissues such as brain astrocytes to increase local concentrations of T_3_ from circulating T_4_. However, we propose that T_4_ may also act directly on TRs to regulate gene transcription in neurons in the absence of deiodinase 2 conversion to T_3_.

Determining the effective cellular concentrations of T_4_ and T_3_ in the brain, or in any tissue, is difficult due to the complexities of TH synthesis, transport, and regulation. Vertebrates have developed multiple mechanisms to ensure delivery of appropriate levels of TH to peripheral tissues such as the brain. These include regulation of secretion of THs from the thyroid into serum (21, 22), control of free versus bound levels of THs determined by reversible binding to serum-binding proteins (22), cell-specific expression of TH cell membrane transporters (23, 24), and finally intracellular deiodination of T_4_ to form T_3_ [(22, 25); Figure 1].

Transplacental TH transfer from maternal to fetal circulation is particularly important in vertebrate CNS development [reviewed by Ref. (26)] to ensure appropriate levels of TH are available to the fetus throughout development (16). Throughout the first trimester when TH levels are solely obtained through maternal transfer, free T_4_ levels are high in the fetus, similar to levels of biologically active T_4_ in adults, whereas fetal concentrations of T_3_ are at least 10× lower than T_4_ (16). Notably, T_3_ levels in the fetal cerebral cortex increase somewhat between 12 and 20 weeks PMA (post-menstrual age) when placental deiodinase 2 levels increase (see below), although maternal serum levels of T_3_ are still low. Both T_4_ and T_3_ in the fetus continue to be transferred from maternal origins through the placenta until half-way through pregnancy when endogenous THs are produced by the fetal thyroid. However, because fetal T_4_ synthesis is elevated over that of T_3_ for several weeks at this time, it is possible that an additional window in development exists where fetal circulating T_4_ is quite high and may act as an active hormone with TRs (16).

Outer ring 5′-monodeiodination via cell-specific deiodinases converts a small fraction of the normal serum T_4_ pool to T_3_ (10, 22). Deiodinase 2 is the primary enzyme responsible for intracellular conversion of T_4_ into T_3_ in most local tissues including brain, whereas deiodinase 1 is found primarily in the liver (25, 27). Deiodinase 2 is only expressed in selected cell types within the CNS: astrocytes and tanycytes. These are both glial cell-derived and are located in the hypothalamus (28–30). The other deiodinase enzyme expressed in the CNS is deiodinase 3, selectively expressed in neurons. Deiodinase 3 inactivates both T_4_ and T_3_ by inner ring deiodination to rT_3_ and T_2_ so as to down-regulate local TH concentrations and protect the neuron from supraphysiological levels of TH. Currently it is believed that astrocytes generate active T_3_ from circulating pro-hormone, T_4_, whereas neurons degrade both T_4_ and T_3_ to inactive rT3 and T2, respectively, and thereby regulate local TH availability within the brain. When levels of TH are low, deiodinase 2 levels in brain increase and contrastingly when there are high levels of TH, deiodinase 3 levels increase (19, 30, 31). This balancing act protects the brain from the detrimental effects of hyper- or hypothyroidism.

**Figure 2:**
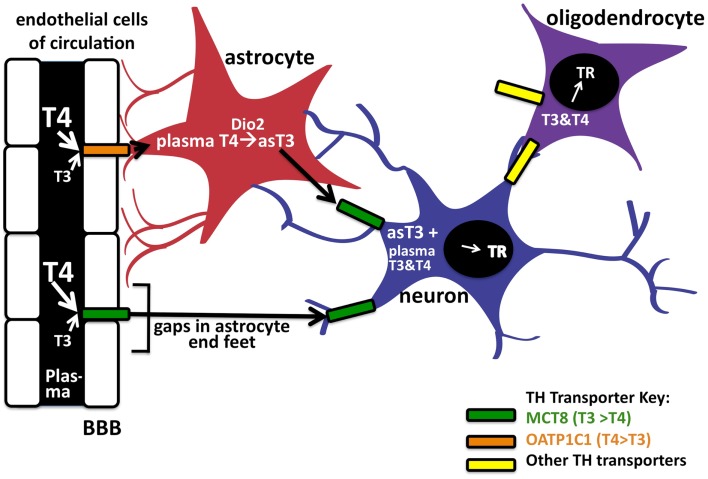
**Entry of TH into brain via the blood–brain barrier**. TH can enter neurons by two pathways. The first is by crossing the endothelial cells of the blood–brain barrier (BBB) by the OAT1P1C transporter to enter astrocyte end feet (in red). After entering astrocytes, T_4_ can be converted into T_3_ via deiodinase 2, to enter the neuron (in blue) by the MCT8 transporter. Circulating T_4_ and T_3_ may also enter neurons (and astrocytes) directly via these transporters through gaps in the astrocyte end feet. Oligodendrocytes (purple), which express TRs, are also known TH cell targets in the CNS. There is also evidence of as-yet unknown TH transporters in the brain; the TH transporters, and their known preferences for T_4_ or T_3_, are indicated in color codes on the right.

T_3_ concentrations equilibrate rapidly in peripheral tissues such as the liver and kidney but appear to take longer to equilibrate in the brain. In general, TH concentrations in the CNS are approximately 20% that of serum concentrations (32); this is likely due to the added complexity of TH transport across the BBB, which is comprised of the endothelial cells of brain capillaries surrounded by astrocyte end feet. To enter the brain, the THs cross the BBB of the choroid plexus via the MCT8 or OATP1C1 TH transporters. T_4_ is thought to predominately enter the CNS in preference to T_3_ as the majority of BBB TH transporters exhibit greater affinities for T_4_ transport [(19, 33); Figure 2]. As mentioned above, after T_4_ is taken up into astrocytes likely by OATP1C1, deiodinase 2 can in turn convert it locally to T_3_. Finally, the astrocyte-generated T_3_ can enter neuronal cells via the MCT8 transporter to bind and activate TRs. Therefore, it is intriguing that the T_4_-activating deiodinase is not expressed in the neurons themselves, where the relevant TRs are located, but in the astrocytes. T_4_ and/or T_3_ also enter the CNS directly via gaps in the end feet of the astrocytes, which do not completely cover the capillaries in contact with the interstitial spinal fluid (34).

## Different TR Isoforms Differ in Their Ability to Bind to T_4_

Thyroid hormones bind TRs, ligand-regulated transcription factors, which bind to specific target DNA sequences and repress or activate target genes through the recruitment and release of accessory proteins. TRs contact their DNA-binding elements as protein dimers, heterodimerizing with another member of the nuclear receptor family, RXRs (primarily Retinoid X Receptors), or homodimerizing with themselves (35–39). TRs exhibit bimodal regulation, typically binding corepressors to repress transcription of target genes in the absence of TH, but releasing corepressors and recruiting coactivators to activate transcription of these “positive response” target genes in the presence of TH (40, 41). These corepressor and coactivator proteins alter the chromatin template or interact with the general transcription machinery to produce the appropriate transcriptional outputs. However, many TR target genes display the opposite properties in that they are expressed in the absence of TH and are repressed in the presence of TH; the molecular mechanisms involved in this “negative response” is not well-understood.

Thyroid hormone receptors are encoded by two distinct genetic loci, denoted THRA and THRB, which are each expressed as alternatively spliced mRNAs to create additional receptor diversity [reviewed in Ref. (42)]. Two of the major TR isoforms are referred to as TRα1 and TRβ1; both bind TH and yet exhibit distinct biological roles [reviewed in Ref. (43)]. TRα1 is expressed early in embryonic development and then widely in adults whereas TRβ1 is expressed later in embryonic development and exhibits a more restricted tissue-expression pattern in adults (31, 44–49). Genetic disruption in mice of TRα1 or TRβ1 indicates that these isoforms have somewhat overlapping, yet distinct roles in normal physiology (45–47, 49, 50).

These two different TR isoforms differ in their ability to respond to T_4_, with TRα1 generally exhibiting a much stronger response to T_4_ than TRβ1. We suggest that different cell types may modulate their relative ability to respond to T_4_ versus T_3_ by altering the relative abundance of different coactivators and corepressors that have distinct responses to T_4_ and T_3_, raising the possibility that T_4_ may be able to function as a direct-acting hormone agonist with TRα1 (Amy C. Schroeder and Martin L. Privalsky, unpublished observations).

## TRα1 Expression in the Brain

Notably, TRα1 encompasses 70–80% of all TR expression in the adult vertebrate brain (2) and TRα1 is present in nearly all neurons (51). Intriguingly, TRα1 is also the predominating TR isoform early in fetal brain development (detected by 8.1 weeks and increasing until 13.9 weeks post-menstrual age). Critical roles in CNS development are known to be mediated by TRα1 including TH-dependent oligodendrocyte differentiation (52). If TRα1 is inactivated, the number of mature oligodendrocytes after T_3_ treatment is decreased (52). The commitment of these cells as oligodendrocytes is therefore believed to be linked to cell-specific TRα1 expression while the availability of TH regulates the timing of differentiation (52). In fact, maturation of several cell types in the brain in development may depend on specific windows of TRα1 expression and involve a complicated interplay between TRs, THs, and coregulators (2). Additionally, TRα1 is known to exhibit important roles in later stages of neurodevelopment and its expression persists in adult neurons. Therefore, it is interesting that expression of the TRα1 isoform predominates in both fetal and in adult brain at the same times when free T_4_ levels appear to be at biologically active levels (16), suggesting windows in brain development may exist where T_4_ may act on TRα1.

## Deiodinase 2-Deficient Mice Exhibit Normal CNS Development and Function

As noted above, deiodinase 2 expression does not overlap TR receptor expression in the brain. Deiodinase 2 is expressed instead in astrocytes whereas the TRs are expressed in neurons along with deiodinase 3 [(28, 29); Figure 2]. The current theory therefore suggests astrocytes are involved with T_4_ uptake from capillaries to subsequently generate a source of locally generated T_3_. Conversion of T_4_ into T_3_ via deiodinase 2 in astrocytes has been estimated to produce as much as 80% of the T_3_ bound to the TRs in the brain (18), suggesting astrocyte deiodinase 2 is important for generating local T_3_ concentrations. Therefore, many argue that deiodinase 2 likely plays a critical role in developing brain by providing the necessary amount of T_3_. If this were in fact the case, one would predict the absence of deiodinase 2 would result in detrimental defects in CNS development similar to that seen in hypothyroidism.

However, the Galton lab produced a deiodinase 2-deficient and a deiodinase 2/deiodinase 1 dual-deficient mouse (KOs) without any evident defects in brain development or function (27, 53). The deiodinase KO mice demonstrated slightly elevated circulating T_4_ and TSH levels, and normal thyroid-secretion of T_3_ but no tissue-level production of T_3_ from T_4_ (27). Notably, these mice did not display any signs of hypothyroidism and have no gross physiological or behavioral abnormalities (27). The deiodinase KO was also combined with an MCT8 TH transporter knockout (54, 55); this combination resulted in minor neuronal defects mostly noted by decreased expression of genes in the neural cortex, which are usually positively regulated by T_3_, however, most neural development and function was normal. KO mice studies suggest that T_3_ transport into the brain and local conversion of T_4_ to T_3_ in the brain are not essential for normal brain function in mice, and suggest that CNS T_3_-defects do not produce syndromes as severe as that seen in the hypothyroid mice (27).

Many suggest that there might be compensation in the deiodinase KO mice through the absorption of more T_3_ directly from circulation via the MCT8 transporter in endothelial cells of the BBB, but it should be again noted that the parallel transporters such as OATP1C1 and OATP2 favor T_4_ transport (56, 57) and it is unlikely that T_3_ can be transported into the brain at rate equivalent to T_4_ transport. We suggest that in the absence of available T_3_, T_4_ can act as an active TH in the brain working on, most likely, TRα1. Interestingly, in the absence of deiodinase 1 and 2, positively regulated TH genes in the cerebral cortex remain unaffected but negatively regulated TH genes appear to be impaired in a way that parallel the hypothyroid mice (27, 58). Perhaps in the absence of deiodinase 2, T_4_ can act as an active hormone in brain cells to activate positively regulated TH genes, but not to repress negatively regulated TH genes.

It should be noted that humans with MCT8 mutations display severe neurodevelopmental defects with psychomotor retardation and abnormal serum TH levels (57, 59). Contrastingly, MCT8 KO mice mimic the human MCT8 mutations in their thyroid phenotype but display no obvious brain developmental defects (57, 59). It is therefore possible that the need for locally produced T_3_, and/or the presence of alternative T3-specific transporters, differ in mice and in humans (55).

## TR Coregulators and the Brain

T_4_ efficiently recruits many coactivators to TRα1, with certain well-established TR coactivators (SRC1 and TRAP220) exhibiting a T_4_ response equal or near equal to that induced by T_3_ (Amy C. Schroeder and Martin L. Privalsky, unpublished data). SRC1 mRNA is expressed in many tissues during development including the CNS (60). TRAP220 is also expressed in the developing brain and is thought to play a regulatory role in the process of cell proliferation and differentiation, in learning, and in memory formation (61). The widespread expression of TRAP220 in the developing brain appears to parallel TRα1 expression. Therefore, CNS development correlates with a high level of expression of TRα1 together with TRAP220 and/or SRC1 and may provide an opportunity for T_4_ to directly regulate gene transcription. CNS cell-specific differences in TR isoform and cofactor levels or function are likely to contribute to differences in T_4_ hormone response and may suggest a means by which the T_4_ sensitivity of a given CNS cell type can be regulated in response to internal or external signals.

## A Possible Direct Role for T_4_ in Brain: Are There Contexts in the Brain in Which T_4_ is a Direct-Acting TRα1 Agonist?

Several recent studies have led to the view that T_4_ exhibits non-genomic roles that do not require conversion to T_3_ (20) but which have not challenged the general view that T_3_, not T_4_, is the only direct, biologically relevant agonist for nuclear TR function. Our own experiments indicate that TRα1 has the potential to act as a dual sensor of both T_4_ and T_3_ (Amy C. Schroeder and Martin L. Privalsky, unpublished observations).

Although the effective concentration of T_4_ in the brain is difficult to determine, it is plausible that T_4_ levels are sufficient to induce activation of TRα1-regulated genes in the brain even in the absence of T_3_. We suggest that the normal mix of T_4_ and T_3_ in the brain may actually confer a mixed T_4_/T_3_ transcription response mediated primarily by TRα1, together with a more pure T_3_ response mediated primarily by TRβ1. Notably, mice in which both deiodinase 1 and 2 have been genetically ablated, and thus lack astrocyte deiodinase conversion of T_4_ to T_3_, display only very mild defects in their physiological with little to no neurological defects (27). If, as indicated by these knockouts, T_4_ is not absolutely required in its traditional role as a pro-hormone, the dominance of T_4_ to T_3_ in the circulation and transport into the CNS may instead reflect a novel role of T_4_ as a direct-acting hormone and this direct role may be helping to ameliorate the effects of the deiodinase knockouts in the CNS.

In conclusion, TH endocrinology in the CNS is tightly regulated at multiple tiers. Negative feedback loops in the hypothalamus and the pituitary control T_3_ and T_4_ output by the thyroid gland itself. Further, multiple phenomenon functions together to modulate the transport of circulating TH through the BBB, and multiple transporters act together to directly alter TH availability in the CNS itself. Additionally, conversion of intracellular T_4_ into T_3_ by deiodinase 2, inactivation of both T_3_ and T_4_ by deiodinase 3, and, the ability of different TR isoforms and different coregulators to respond directly to T_4_ versus T_3_ further regulate the CNS response to TH. Operating together, we propose these mechanisms serve to maintain proper endocrine homeostasis while permitting the CNS to respond to developmental and physiological needs.

## Conflict of Interest Statement

The authors declare that the research was conducted in the absence of any commercial or financial relationships that could be construed as a potential conflict of interest.
